# Improving visibility of small anatomical details on low and ultra-low dose computed tomography with artificial intelligence-based image reconstructions

**DOI:** 10.1093/rpd/ncaf171

**Published:** 2026-03-13

**Authors:** Micael Oliveira Diniz, Åse A Johnsson, Rauni Rossi Norrlund, Jenny Vikgren, Walter Cifuentes Ramirez, Sara Ku, Magnus Båth, Angelica Svalkvist

**Affiliations:** Department of Radiology, Institute of Clinical Sciences, Sahlgrenska Academy, University of Gothenburg, 41345 Gothenburg, Sweden; Department of Radiology, Sahlgrenska University Hospital, 41346 Gothenburg, Region Västra Götaland, Sweden; Department of Radiology, Forth Valley Royal Hospital, NHS Forth Valley, Larbert FK5 4WR, Scotland, United Kingdom; South East Scotland Breast Screening Service, Edinburgh EH11 2JL, Scotland, United Kingdom; Department of Radiology, Institute of Clinical Sciences, Sahlgrenska Academy, University of Gothenburg, 41345 Gothenburg, Sweden; Department of Radiology, Sahlgrenska University Hospital, 41346 Gothenburg, Region Västra Götaland, Sweden; Department of Radiology, Institute of Clinical Sciences, Sahlgrenska Academy, University of Gothenburg, 41345 Gothenburg, Sweden; Department of Radiology, Sahlgrenska University Hospital, 41346 Gothenburg, Region Västra Götaland, Sweden; Department of Radiology, Institute of Clinical Sciences, Sahlgrenska Academy, University of Gothenburg, 41345 Gothenburg, Sweden; Department of Radiology, Sahlgrenska University Hospital, 41346 Gothenburg, Region Västra Götaland, Sweden; Department of Radiology, Sahlgrenska University Hospital, 41346 Gothenburg, Region Västra Götaland, Sweden; Department of Radiology, Sahlgrenska University Hospital, 41346 Gothenburg, Region Västra Götaland, Sweden; Department of Medical Radiation Sciences, Institute of Clinical Sciences, Sahlgrenska Academy, University of Gothenburg, 41345 Gothenburg, Sweden; Department of Biomedical Engineering and Medical Physics, Sahlgrenska University Hospital, 41345 Gothenburg, Region Västra Götaland, Sweden; Department of Medical Radiation Sciences, Institute of Clinical Sciences, Sahlgrenska Academy, University of Gothenburg, 41345 Gothenburg, Sweden; Department of Biomedical Engineering and Medical Physics, Sahlgrenska University Hospital, 41345 Gothenburg, Region Västra Götaland, Sweden

## Abstract

To assess how computed tomography (CT) image reconstruction techniques affect perceived diagnostic image quality at varying radiation dose levels in chest imaging. A PBU-50 anthropomorphic phantom (small adult-sized model) and an air-dried human lung specimen were scanned on the same CT system (Revolution Apex™, GE Healthcare) at six dose levels (CTDI_vol_) from 0.07 to 2.19 mGy for the smallest phantom size. Images were reconstructed using deep learning image reconstruction-high (DLIR-H), adaptive statistical iterative reconstruction at 40 per cent (ASiR-V), and filtered back projection (FBP). Five radiologists assessed anatomical reproduction, noise, artefacts, and diagnostic quality using ViewDEX. Descriptive statistics and visual grading characteristics analysis were used. In general, DLIR-H scored higher than ASiR-V and FBP. While maintaining image quality, DLIR-H allowed dose reduction compared to FBP. All methods were deemed acceptable for diagnosing pulmonary nodules, fibrosis, and peribronchial pathology. The results indicate that DLIR-H improves image quality in comparison to FBP and ASiR-V and may enable radiation dose reduction while maintaining clinical image quality.

## Introduction

Computed tomography (CT) of the chest is a gold standard method of modern thoracic imaging, widely used to visualize anatomical structures and detect pathologies. Nevertheless, this diagnostic method is associated with ionizing radiation exposure of the patients [1]. This has driven ongoing efforts to reduce radiation dose without compromising diagnostic image quality.

The effective dose for a standard clinical CT is generally in the order of 2–7 mSv [2] Regarding low-dose CT (LDCT) and ultra-low-dose CT (ULDCT) there are no strict definitions. However, effective doses about 1.5 mSv are commonly regarded as LDCT [3], while effective doses below 0.5 mSv may be considered as ULDCT. ULDCT may even include effective doses comparable to chest X-ray (0.05 mSv) [4].

Different LDCT and ULDCT protocols have emerged as possible solutions, particularly in screening and follow-up scenarios [5–8]. Still, one of their limitations is a possible decrease in image quality associated with reduced radiation doses, particularly when visualizing small anatomical details crucial for clinical diagnosis. As so, multiple reconstruction techniques have been developed with the purpose to overcome these limitations. Two of these reconstruction techniques are adaptive statistical iterative reconstruction (ASiR-V) (GE Healthcare, Waukesha, USA) and deep learning image reconstruction (DLIR), commercially known as TrueFidelity™ (GE Healthcare, Waukesha, USA). These two techniques have been shown to present better diagnostic image quality when compared to filtered back projection (FBP) at comparable radiation dose levels [9, 10].

FBP delivers accurate image results but struggles with high image noise and artefacts in low-dose scenarios [11]. Additional limitations with FBP include challenges in handling deviations from the ideal CT system model, which can compromise image quality. Examples of such limitations are related to X-ray physics (i.e. beam hardening and scatter), the statistical nature of data (i.e. low X-ray photon flux and electronic noise), and system geometry factors (i.e. partial volume effects, detector cell size, and finite X-ray focal spot size). As a result of these constraints, achieving acceptable image quality often requires higher radiation doses, increasing patient risk. Alternatively, reconstructed images may exhibit reduced quality, potentially impacting diagnostic accuracy [9]. Consequently, iterative reconstructions (IRs) were developed for CT imaging to address the limitations inherent in FBP [12]. The benefit of IR over FBP is that it reduces noise in the images, which enables dose reduction without substantial reduction in diagnostic image quality.

The first IR algorithm commercially introduced to offer substantial noise reduction for diagnostic CT imaging was adaptive statistical iterative reconstruction (ASiR) [12]. The more advanced ASiR-V enhances the capabilities of ASiR by incorporating advanced noise and object modelling along with added physics-based modelling. These improvements enable ASiR-V to further reduce image noise in CT imaging while maintaining or even enhancing its diagnostic quality, facilitating low-contrast detectability when compared to FBP [12]. However, when employed at full strength, it still faces challenges related to image texture stemming from the limited complexity of its underlying model [11]. The reconstructed images frequently deviate from the visual characteristics of those produced by FBP under ideal conditions, primarily due to the level of modelling complexity the algorithm can accommodate. This deviation is often described as resulting in images textures that appear ‘plastic-looking’ ‘blotchy’, or ‘artificial’ [13].

The multiple challenges related to FBP, and ASiR-V led to the next development on image reconstruction, deep learning image reconstruction-high (DLIR-H). DLIR leverages a deep neural network to overcome limitations associated with traditional IR methods. The deep neural network is trained using FBP datasets, enabling it to separate useful image data signals from noise. This allows for intelligent noise suppression while preserving anatomical structures [11]. As a result, the noise can be minimized while the spatial resolution and contrast can be enhanced, without altering the noise texture [14, 15]. Previous studies have shown that DLIR outperformed FBP and ASiR-V in terms of diagnostic image quality in LDCT and ULDCT of the chest [4, 16].

In this study, it was hypothesized that the use of DLIR, instead of FBP or ASIR-V, would enable a significant reduction in radiation dose while maintaining diagnostic image quality and visibility of small anatomical details in CT images. Consequently, the aim of the present study was to investigate image quality at various dose levels using these different image reconstruction methods.

## Materials and methods

### Scanned material

The PBU-50 anthropomorphic phantom [17], a full body small adult human-size phantom developed by Kyoto Kagaku (Kyoto, Japan), including among others, synthetic lungs, mediastinum, and skeleton was used in the present study. The phantom comes with additional body plates that can be used to simulate different body mass index (BMI). PBU-50 without plates corresponds approximately to a patient with BMI 19 (Size 1). PBU-50 with two thinner plates (one on the front of the phantom and one on the back) corresponds to approximately BMI 32 (Size 2). PBU-50 with two thicker plates (one on the front and one on the back) corresponds to approximately BMI 40 (Size 3).

Additionally, a formalin fixed, air dried, inflated historical specimen of human lungs was used in the study to evaluate image quality for the diagnosis of pulmonary nodules, peribronchial pathology, and fibrosis, which were not represented in the PBU-50 phantom.

At least five pulmonary nodules were present in the specimen along with one area of fibrosis, and two regions of peribronchial pathology. The specimen was donated to the radiology department at Sahlgrenska University Hospital by Professor Emeritus Ulf Tylén (University of Gothenburg, Sweden).

### Image acquisition

The PBU-50 phantom (Sizes 1–3) and the historical lung specimen were scanned on the same CT-scanner (Revolution CT APEX Edition, GE Healthcare, Milwaukee, USA) in an antero-posterior supine position.

Both the PBU-50 phantom and the lung specimen were examined using six different examination protocols at various dose levels, ranging from a volume computed tomography dose index (CTDI_vol_) of 0.07 mGy to a CTDI_vol_ of 9.83 mGy, as shown in Table 1. All examinations were performed on the same day.

**Table 1:** The examination parameters for the six protocols (P0 to P5), where P0 = standard full dose protocol, P1 = low dose protocol 1, P2 = low dose protocol 2, P3 = low dose protocol 3, P4 = low dose protocol 4, P5 = low dose protocol 5 (ultra-low dose protocol).

Protocol	P0	P1	P2	P3	P4	P5
Scan type	Helical	Helical	Helical	Helical	Helical	Helical
SFOV	Large body	Large body	Large body	Large body	Large body	Large body
Collimator (mm)	80	80	80	80	80	80
Rotation time (s)	0.5	0.5	0.5	0.5	0.28	0.28
Pitch	0.992	0.992	0.992	1.375	1.531	1.531
Slice thickness—scanned (mm)	0.625	0.625	0.625	0.625	1.25	1.25
Slice thickness—reconstructed (mm)	1.25	1.25	1.25	1.25	1.25	1.25
Noise index	28	32.5	45	65	85	85
Tube voltage (kV)	120	120	120	120	120	120
AEC min–max range (mA)	60–560	10–560	20–110	10–110	10–110	10–15
Convolution kernel	Standard	Standard	Standard	Standard	Standard	Standard
ODM	No	No	No	No	No	No
CTDI_vol_ (mGy)						
PBU-50						
size 1	2.19	1.31	0.74	0.29	0.13	0.07
size 2	3.89	2.66	1.20	0.47	0.17	0.07
size 3	9.83	6.33	2.37	0.84	0.26	0.11
Lung specimen	2.04	0.68	0.34	0.24	0.12	0.07

AEC min–max range = automatic exposure control minimal and maximal range; CTDI_vol_ = computed tomography dose index; ODM = organ dose modulation; P = protocol; SFOV = scanned field of view.

### Image reconstruction

**Figure 1:**
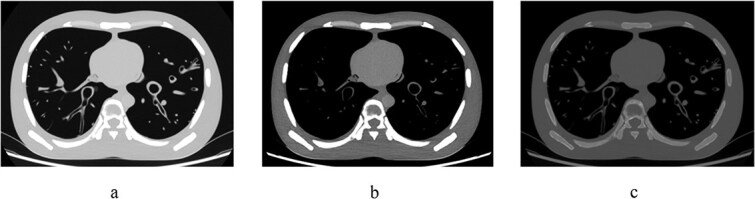
Transverse computed tomography images of the PBU-50 phantom (Size 1, without additional plates), acquired at a full radiation dose (protocol 0) and reconstructed using deep learning image reconstruction-high (DLIR-H), are illustrated with the following windowing settings: (a) lung windowing, (b) mediastinum windowing, (c) bone windowing.

**Figure 2:**
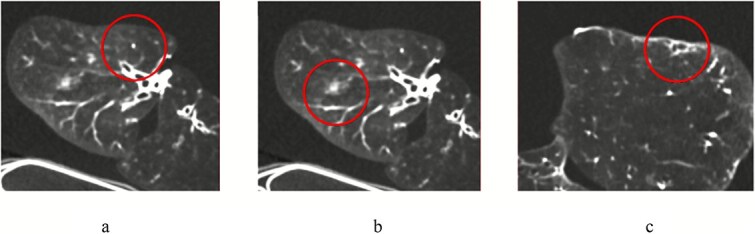
Transverse computed tomography images of the historical lung specimen, acquired at a full radiation dose (protocol 0) and reconstructed with deep learning image reconstruction-high (DLIR-H), showing the following findings highlighted by a circle: (a) lung nodule, (b) peribronchial pathology (tree-in-bud/ground-glass), and (c) lung fibrosis.

The raw image data from the examinations were reconstructed using three different techniques: FBP, ASiR-V at 40 per cent, and DLIR-H, producing 54 unique image series of the PBU-50 phantom (three sizes at six different examination protocols and three different image reconstructions) and 18 image series of the historical lung specimen (six different examination protocols and three different image reconstructions). Illustrations of the scanned phantom and the lung specimen are given in Figs 1 and 2.

### Image quality evaluation

**Figure 3:**
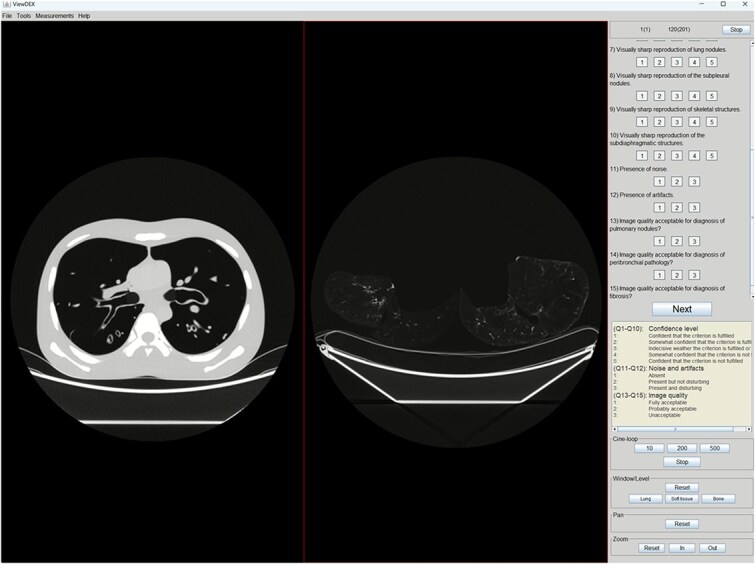
Monitor setup as presented to the observers, showing transverse computed tomography (CT) images of the PBU-50 phantom (Size 1, without additional plates) on the left and transverse CT images of the lung specimen on the right, both acquired at full radiation dose (protocol 0) and reconstructed using deep learning image reconstruction-high (DLIR-H).

Five radiologists with different levels of experience in the field of thoracic radiology evaluated the reconstructions. Three with subspecialization in thoracic radiology, each with more than 25 y of experience, and two with consultant level but no subspeciality and ~6 y of general radiology experience each. Image evaluation was performed using ViewDEX [18–21]. An example of monitor setup as presented to the observers is shown in Fig. 3. Observers were presented with simultaneous images of the phantom and the lung specimen for each examination protocol and reconstruction method. The image reconstructions were presented in randomized order to the observers, with a unique randomization sequence for each reviewer in order to avoid possible biases due to observer learning curves in the beginning of the study. Since the study included three phantom sizes but only one lung specimen, the same lung specimen images were paired with each of the three phantom sizes for a given examination protocol and reconstruction method. For example, lung specimen images acquired with Protocol 1 and reconstructed using FBP were each separately paired and evaluated alongside phantom images of Sizes 1–3, which were also acquired using Protocol 1.

The radiologists answered fifteen questions (Table 2) divided in three main categories: reproduction of anatomy (Questions 1–10), noise and artefacts (Questions 11–12), and overall image quality (Questions 13–15). Questions 13–15 were derived from previous work [4]. The observers were instructed to answer Questions 1–10 based on the phantom, Questions 11–15 based on both the phantom and the specimen. Regarding the phantom, suggested anatomical structures for Questions 1–10 were marked in images provided in the instructions to the observers.

**Table 2:** The 15 image quality questions and the possible answer alternatives divided by three categories (reproduction of anatomy, noise and artefacts, image quality).

Question category	Question	Answer alternatives
Reproduction of anatomy	1. Sharp reproduction of the intrathoracic airfield. 2. Visually sharp reproduction of aorta descending aorta at the level of the carina. 3. Visually sharp reproduction of large and medium sized pulmonary vessels at hilus. 4. Clear reproduction of a right inferior vessel 3 divisions on axial plane (left if right one is not visible). 5. Clear reproduction of 3 subdivisions on axial plane of an apical bronchus on the right size (left if right one is not visible). 6. Visually sharp reproduction of the border between pleura and the thoracic wall. 7. Visually sharp reproduction of lung nodules. 8. Visually sharp reproduction of the subpleural nodules. 9. Visually sharp reproduction of skeletal structures. 10. Visually sharp reproduction of the subdiaphragmatic structures.	1. Confident that the criterion is fulfilled 2. Somewhat confident that the criterion is fulfilled 3. Indecisive as to whether the criterion is fulfilled or not 4. Somewhat confident that the criterion is not fulfilled 5. Confident that the criterion is not fulfilled
Noise and artefacts	11. Presence of noise. 12. Presence of artefacts.	1. Absent 2. Present but not disturbing 3. Present and disturbing
Image quality	13. Is the image quality acceptable for diagnosis of pulmonary nodules? 14. Is the image quality acceptable for diagnosis of peribrochial pathology? 15. Is the image quality acceptable for diagnosis of fibrosis?	1. Acceptable 2. Probably acceptable 3. Unacceptable

### Data analysis

The results from the observer study were analysed using descriptive statistics and visual grading characteristics (VGC) analysis [22]. The relationship between phantom size and the reproduction of anatomical structures, image noise, artefacts, and overall image quality was not assessed, as this was beyond the primary scope of the present study.

The data obtained from the study were analysed in two different parts.

#### Data analysis—Part 1

To analyse the first 10 questions regarding reproduction of anatomy, a VGC analysis was conducted, with the goal to explore the optimal reconstruction settings for visualization of different sized structures at different examination protocols. This method is non-parametric and rank-invariant, allowing for a comparison of visual grading data between two different imaging conditions [22].

The VGC Analyzer software [23] was used to calculate the area under the VGC curve (AUC_VGC_) and conduct statistical analysis of the VGC data. The AUC_VGC_ is calculated using both the trapezoidal rule and binormal curve fitting. The statistical analysis accommodates both paired and unpaired data, considering both fixed and random reader scenarios.

The VGC analysis in the present study was based on a random reader assumption and the trapezoidal rule of curve fitting. To assess the uncertainty of the AUC_VGC_, bootstrapping of the data is applied. The random reader analysis also involves bootstrapping of observers, allowing the findings to be generalized to a broader population.

An AUC_VGC_ value below 0.5 suggests that the reference condition received higher ratings, whereas a value above 0.5 indicates that the test condition was rated higher. If the confidence interval (CI) of the AUC_VGC_ includes 0.5, there is no statistically significant difference in ratings between the conditions [22].

The analysis of the data concerning reproduction of anatomy was divided into three subparts.

##### Data analysis—Part 1, Subpart 1

In the first subpart, the 10 questions regarding anatomical reproduction were analysed independently of the examination protocol (all protocols analysed together) using VGC analysis.

##### Data analysis—Part 1, Subpart 2

In the second subpart, the same 10 questions regarding anatomical reproduction were analysed individually for each examination protocol using VGC analysis. In the first and second subparts, FBP, for each examination protocol, was the reference condition and ASiR-V and DLIR-H were the test conditions. Additionally, DLIR-H was compared to ASiR-V, in these cases ASiR-V was the reference condition and DLIR-H was the test condition.

##### Data analysis—Part 1, Subpart 3

In the third subpart, the standard full dose protocol (P0) reconstructed with FBP was used as reference in order to evaluate how much the radiation dose possibly can be reduced when replacing FBP with ASIR-V or DLIR-H. The reference P0 FBP was compared to ASiR-V and DLIR-H for each of the six different protocols.

#### Data analysis—Part 2

The questions concerning noise and artefacts (Questions 11 and 12) as well as questions related to diagnostic image quality (Questions 13–15) were evaluated using descriptive statistics. For each examination protocol, all three evaluations of the lung specimen were included.

## Results

### Reproduction of anatomy–Part 1

When evaluating the preferred reconstruction for assessing reproduction of anatomy (Questions 1–10) for all six protocols combined (Subpart 1), the comparison of DLIR-H with FBP and ASiR-V resulted in an AUC_VGC_ of 0.62 (CI: 0.58–0.67) and 0.58 (CI: 0.54–0.62), respectively. Hence, DLIR-H was the preferred reconstruction method.

**Figure 4:**
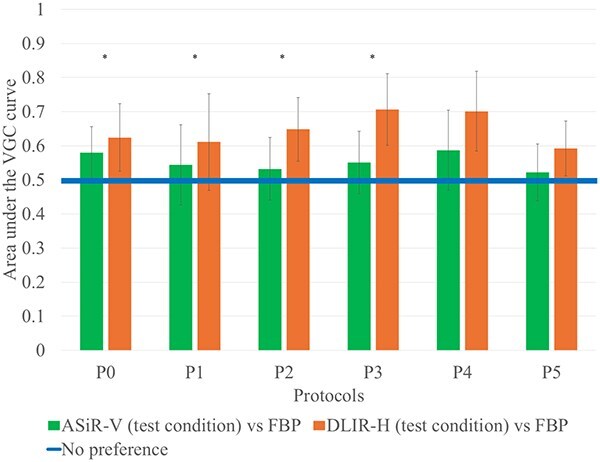
The results from the visual grading characteristics (VGC) analysis of questions 1–10 (for each examination protocol), using filtered back-projection (FBP) as the reference condition, where the horisontal line represents an area under the VGC curve (AUC_VGC_) of 0.5 (no difference), the error bars represent the 95% confidence intervals of the AUC_VGC_, and ‘*’ indicates a statistically significant difference between adaptive statistical iterative reconstruction-V (ASiR-V) and deep learning-based image reconstructions-high (DLIR-H) for each protocol.

The results from Subpart 2 are shown in Fig. 4. When comparing the reconstructions (DLIR-H, ASiR-V, and FBP) per protocol, DLIR-H was preferred over FBP in all protocols with statistically significant difference for all the protocols except protocol P1. Regarding ASiR-V no statistically significant difference was found compared to FBP. Concerning the comparison between DLIR-H and ASiR-V, there was a statistically significant advantage for DLIR-H for protocols P0-P3.

**Figure 5:**
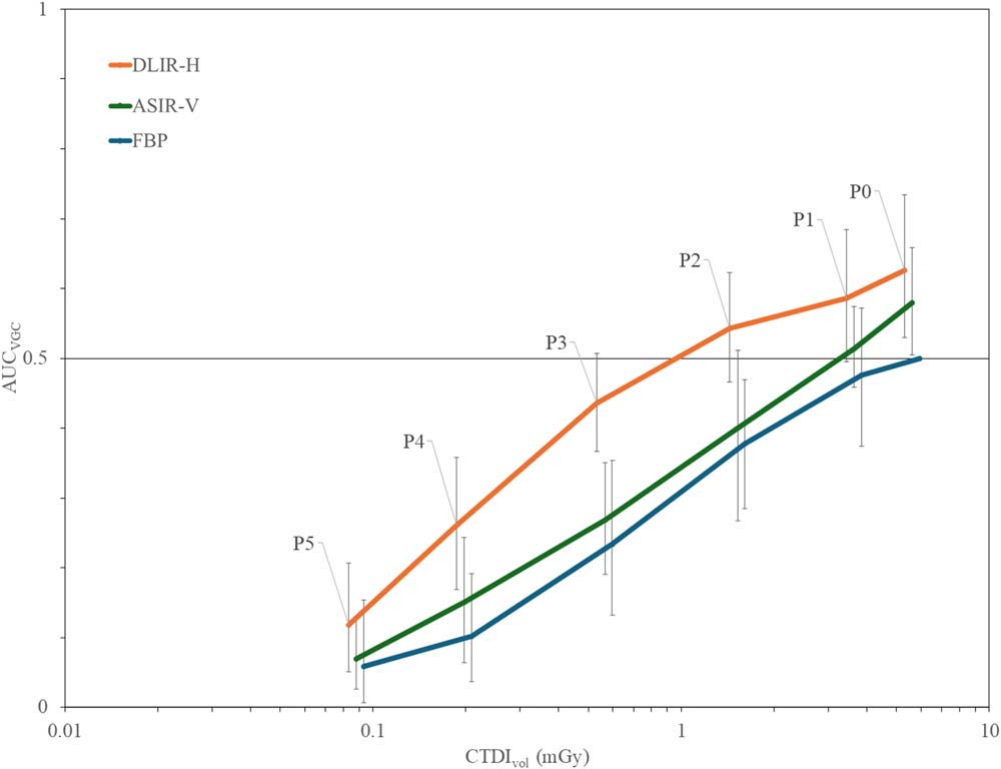
The results from the analysis in which all reconstructions (filtered back-projection (FBP), adaptive statistical iterative reconstruction-V (ASiR-V), and deep learning–based image reconstruction-high (DLIR-H)) were compared to the standard full-dose protocol (protocol 0) reconstructed with FBP (reference condition) across six examination protocols (protocols 0–5) for the 10 questions regarding reproduction of anatomy, where the horisontal line represents an area under the VGC curve (AUC_VGC_) of 0.5 (no difference), and a mean volume computed tomography dose index (CTDI_vol_) was calculated per protocol based on the three phantom sizes (X-axis). (Note: Although all reconstruction methods were originally evaluated at the same radiation dose levels, the results are displayed with slight offsets applied to the CTDI_vol_ values – 6% added for ASiR-V and 12% for FBP at each radiation dose level to visually separate the confidence intervals and avoid overlap in the graph.

In Subpart 3, where DLIR-H, ASiR-V, and FBP were compared to the reference (P0 FBP), DLIR-H was rated higher than or without any statistically significant difference in comparison to P0 FBP for protocols P0–P3.The corresponding results for ASiR-V showed that ASiR-V was rated higher than or without any statistically significant difference in comparison to P0 FBP for protocols P0–P2, as illustrated in Fig. 5. No statistically significant difference was found between P1 FBP and P0 FBP.

### Noise, artefacts, and image quality—Part 2

**Figure 6:**
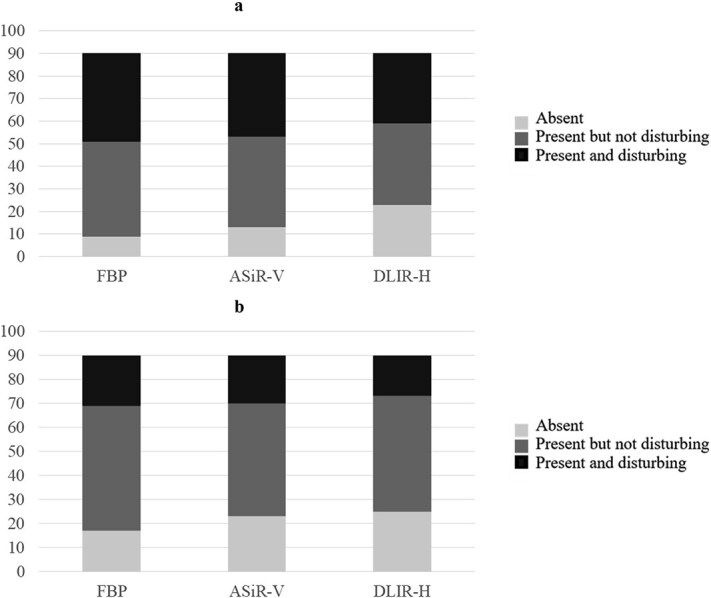
The observers’ responses to (a) Question 11 ‘Presence of noise’ and (b) Question 12 ‘Presence of artefacts’ across all reconstruction methods, without dividing them by examination protocol, where each column represent the number of ratings in each category – absent (light grey), present but not disturbing (medium grey), and present and disturbing (dark grey) for the three image reconstruction techniques (filtered back projection (FBP), adaptive statistical iterative reconstruction-V (ASiR-V), and deep learning image reconstruction-high (DLIR-H)).

In Fig. 6, the distributions of observer ratings regarding noise (a) and artefacts (b) for all examination protocols combined, indicate that the use of ASiR-V and DLIR-H results in a decrease in perceived noise and artefacts.

**Figure 7:**
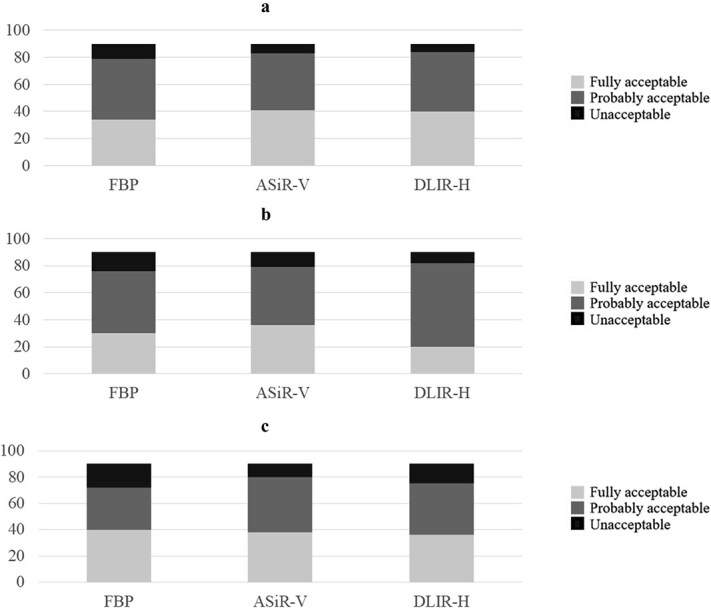
The observers’ responses to (a) Question 13 ‘Is the image quality acceptable for the diagnosis of pulmonary nodules?’, (b) Question 14 ‘Is the image quality acceptable for the diagnosis of peribronchial pathology?’, and (c) Question 15 ‘Is the image quality acceptable for the diagnosis of fibrosis?’, for all examination protocols (protocols 0–5) combined, where each column represent the number of ratings in each acceptability category – fully acceptable (light grey), probably acceptable (medium grey), and unacceptable (dark grey) for the three image reconstruction techniques (filtered back projection (FBP), adaptive statistical iterative reconstruction-V (ASiR-V), and deep learning image reconstruction-high (DLIR-H)).

For the diagnosis of pulmonary nodules, peribrochial pathology, and fibrosis, all reconstruction methods were in the large majority of the cases rated as fully acceptable or probably acceptable, for all protocols combined, as shown in Fig. 7.

Regarding the individual protocols, it was observed that image quality ratings deteriorated with decreasing dose levels.

**Figure 8:**
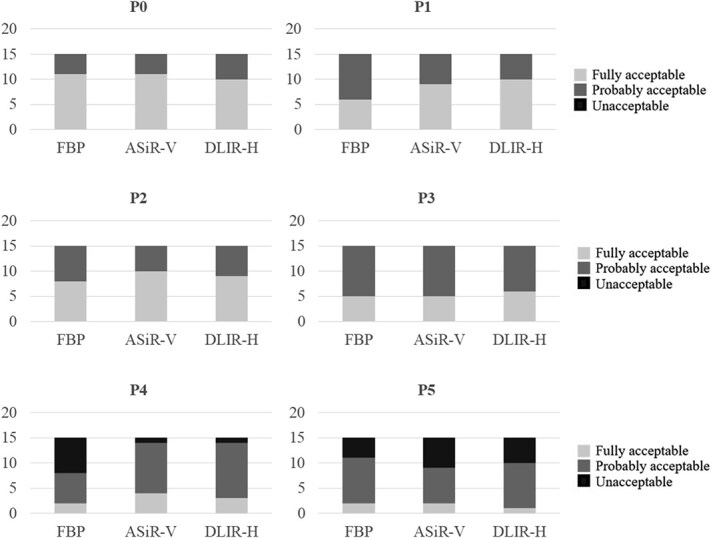
The observers’ responses to Question 13, ‘Is the image quality acceptable for the diagnosis of pulmonary nodules?’, for different examination protocols (P0-P5), where each column represent the number of ratings in each acceptability category – fully acceptable (light grey), probably acceptable (medium grey), and unacceptable (dark grey) for the three image reconstruction techniques (filtered back projection (FBP), adaptive statistical iterative reconstruction-V (ASiR-V), and deep learning image reconstruction-high (DLIR-H)).

The diagnosis of pulmonary nodules appeared to be relatively unaffected by dose reductions down to protocol P3 (Fig. 8). Only in protocol P4 a beneficial effect of DLIR-H could be found.

**Figure 9:**
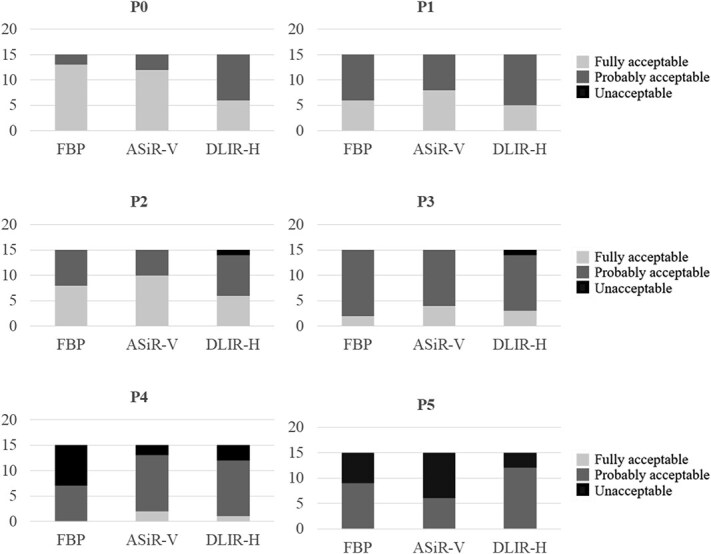
The observers’ responses to Question 14, ‘Is the image quality acceptable for the diagnosis of peribronchial pathology?’, for different examination protocols (P0-P5), where each column represent the number of ratings in each acceptability category – fully acceptable (light grey), probably acceptable (medium grey), and unacceptable (dark grey) for the three image reconstruction techniques (filtered back projection (FBP), adaptive statistical iterative reconstruction-V (ASiR-V), and deep learning image reconstruction-high (DLIR-H)).

**Figure 10:**
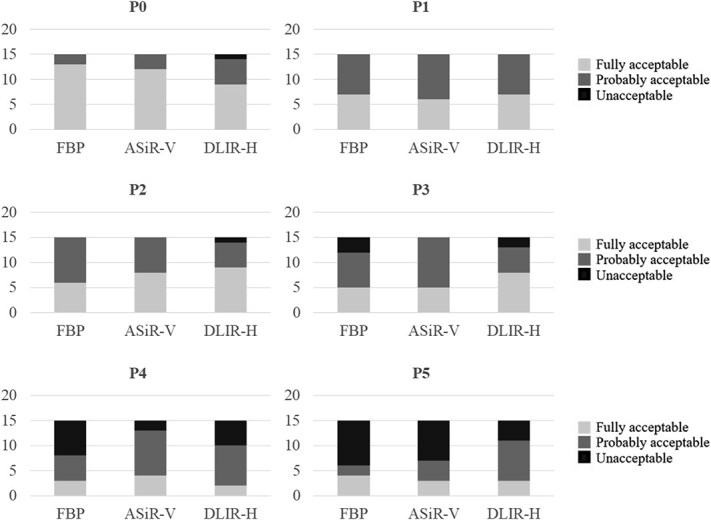
The observers’ responses to Question 15, ‘Is the image quality acceptable for the diagnosis of fibrosis?’, for different examination protocols (P0-P5), where each column represent the number of ratings in each acceptability category – fully acceptable (light grey), probably acceptable (medium grey), and unacceptable (dark grey) for the three image reconstruction techniques (filtered back projection (FBP), adaptive statistical iterative reconstruction (ASiR-V), and deep learning image reconstruction-high (DLIR-H)).

Concerning peribrochial pathology and fibrosis, there was no apparent benefit in image quality with the DLIR-H reconstruction method (Figs. 9 and 10).

## Discussion

As hypothesized, DLIR-H demonstrated the best performance related to anatomical reproduction, aligning with findings from previous studies [11]. Similarly to the present study, and despite employing a different type of DLIR for post-processing images reconstructed with ASiR-V, Ye *et al*. demonstrated that DLIR enhances overall image quality, improves nodule detection rates, increases nodule edge sharpness, and yields more accurate measurements of pulmonary nodules when applied to ULDCT [24].

When using FBP at standard examination protocol (P0) as a reference for image quality, it was observed that DLIR-H might enable dose reduction while maintaining perceived diagnostic image quality when compared to FBP and ASiR-V. These results also align with prior studies [11], including a patient study by Svalkvist *et al*. [4], in which ASiR-V and DLIR-H were evaluated in both standard-dose and ULDCT. However, the present results should be interpreted with caution, as the results are based on a small sample of images which leads to large CIs for the comparisons. Despite that higher radiation dose reduction can be obtained with DLIR-H followed by ASiR-V, a radiation dose reduction may also be achieved with FBP Consequently, the relative dose reductions reported for ASiR-V and DLIR-H become less pronounced when considering that FBP itself allows for dose lowering, as the comparisons are made against a general reference (P0 FBP) rather than a minimum dose required to achieve equivalent image quality with FBP.

Previous studies have shown that that the use of DLIR-H allowed a decrease in noise [14, 16] compared to the traditional FBP and to ASiR-V. The findings in the present study are in line with those studies.

As expected, there was an increasing presence of artefacts in protocols using lower radiation dose, and slightly less artefacts were reported regarding DLIR-H. It should be noted that there was no prior discussion among the observers to establish a standardized definition of an artefact. Their assessments were based solely on their clinical experience in image interpretation.

FBP, ASiR-V, and DLIR-H were in most of the cases acceptable for the diagnosis of pulmonary nodules, peribrochial pathology, and fibrosis. These results reflect the fact that all three methods are approved for diagnostic purposes [9, 11, 12]. Moreover, diagnostic image quality deteriorated with decreasing dose levels, and as expected. The advantage of using DLIR over ASIR-V 40 per cent for image reconstruction appears to be greater with the lower dose protocols compared to the full-dose protocol, these results are in accordance with a previous study by Svalkvist *et al*. [4]. The higher dose sensitivity observed for the diagnosis of fibrosis compared to nodule detection may be attributed to the fact that clinicians are accustomed to identifying nodules using other imaging modalities, such as tomosynthesis, even at lower radiation doses. In contrast, the assessment of fibrosis typically relies on higher radiation dose techniques and high-resolution CT [25], which may heighten sensitivity to dose-related image quality changes in its diagnosis. Furthermore, ground-glass opacities (GGOs) can be an early sign of focal fibrosis, especially when remaining over time and appearing alongside other signs such as reticulation or traction bronchiectasis [25–27]. Ichikawa *et al*. previously demonstrated that CTDI_vol_ is the primary determinant of GGO visibility across different reconstruction kernels and slice thickness variations [28]. This further emphasizes that the detection of early fibrotic changes, such as GGO, may require higher radiation dose levels to ensure adequate diagnostic accuracy.

Anthropomorphic phantoms are commonly used in diagnostic imaging research, especially in studies aiming at comparing the effects of different image reconstructions on image quality. One of the benefits of using phantoms is that unnecessary radiation exposure of individuals is avoided. Many previous studies on DLIR have therefore been conducted using anthropomorphic phantoms. For example, in a similar fashion to the present study, Racine *et al*. performed a task-based evaluation using a phantom to characterize the performance of a deep learning reconstruction algorithm, comparing it against FBP and a partial model-based IR method in abdominal CT, demonstrating that, in contrast to ASiR-V, DLIR significantly reduces noise while preserving noise texture and providing a slight improvement in spatial resolution. DLIR also surpassed ASiR-V by improving the detectability of both low- and high-contrast simulated abdominal lesions at all tested dose levels [15]. Solomon *et al*. carried out a phantom study to examine the noise characteristics and spatial resolution performance of a DLIR-based CT reconstruction algorithm in thoracic CT. The results from the study indicated that deep learning-based CT reconstruction reduced noise compared to FBP while maintaining noise texture and high-contrast resolution. However, it introduced nonstationary noise in lung textured backgrounds and slightly reduced low-contrast resolution, similar to IR methods [29]. Also Greffier *et al*. employed a phantom to investigate both image quality and potential for radiation dose reduction using a deep learning-based image reconstruction algorithm in CT imaging, showing that DLIR reduced image noise and enhanced spatial resolution and detectability while preserving noise texture [14]. Later, Yang *et al*. conducted a study to evaluate the image quality of DLIR relative to ASIR-V and FBP using phantoms, including thoracic models, focusing on noise, spatial resolution, and overall fidelity, demonstrating that DLIR produced images with FBP-like noise texture and added edge enhancement, while maintaining noise levels similar to ASIR-V [30]. Furthermore, anatomical specimens have previously been used to evaluate the reproduction of lung pathology and image quality on CT. Among them, Sumikawa *et al*. investigated HRCT image quality using autopsied human lungs and compared helical scanning with conventional step-and-shoot techniques, finding that using the appropriate helical mode provide the same quality and diagnostic effectiveness as those obtained with the axial mode [31]. Also, Hata *et al*. assessed the impact of reconstruction matrix size on ultra-high-resolution CT using phantoms and cadaveric lungs, concluding that a larger matrix size improved spatial resolution and image quality, despite increased noise compared to a 512 matrix size [32].

Even though the above-mentioned studies are based on phantom evaluations, a major limitation of the present study is the use of phantoms instead of real patient data. However, it should be noted that the results are in line with the study by Svalkvist *et al.* [4], which was performed on patient data.

Beside the fact that the use of a phantom and a historical specimen instead of real patients might limit the possibilities to generalize of the results to clinical practice, it also limits the possibility to obtain a large image material. Although the PBU-50 phantom could be combined with four additional plates to simulate two more phenotypes based on BMI, the statistical analyses were constrained by the availability of only one phantom and one historical lung specimen. As a consequence, the impact of phantom size on the reproduction of anatomical details, image noise, artefact presence, and overall image quality was not investigated due to the limited statistical data. However, previous research has demonstrated that patient BMI significantly influences these parameters in cardiothoracic imaging. For example, Lee *et al*. demonstrated that image quality on ULDCT of the chest is influenced by the patient’s BMI [33], while Paul *et al*. indicated a notable correlation between chest wall composition and image noise in cardiac CT imaging [34]. Furthermore, a key limitation of this study is the interpretation of AUC values without fully accounting for statistical uncertainty. Conclusions regarding dose reduction based on ‘without statistically significant difference’ results rely heavily on CIs, which, in the context of small datasets, may be wide and include 0.5 regardless of the actual AUC. This increases the risk of overinterpretation, as statistical non-significance does not imply equivalence. Therefore, the interpretation of Fig. 5 should be approached with caution.

It should also be noted that upon scanning, due to the very delicate nature of the lung specimen, it was not surrounded by any additional structures, leading to a significant size difference between the lung specimen and the PBU-50 phantom. Therefore, superiorly perceived image quality could be expected for the lung specimen compared to the images of the PBU-50 phantom when using the same scanning protocol.

An alternative to physical phantoms and anatomical specimens is the use of digital phantoms. These are computer-generated models of human anatomy that mimic the radiological and physical characteristics of real tissues, allowing imaging experiments to be conducted in a controlled and reproducible manner without subjecting real patients to radiation or other risks [35]. This method enables systematic assessment of image quality and reconstruction algorithms under standardized conditions [36]. For the present study, we did however not have access to digital phantoms.

Nonetheless, the study also had several strengths. For example, it involved a fair number of observers, and the use of a phantom and a specimen allowed for consistent scanning at identical radiation dose levels. This approach eliminated concerns related to ionizing radiation exposure and its risks. Furthermore, it reduced interindividual variations, which is often present in human-based research.

## Conclusion

Using realistic physical phantom and lung specimen images, this study indicates that substituting FBP and ASIR-V with DLIR-H may result in improved visual reproduction of anatomical structures and might enable dose reduction without the penalty of reduced diagnostic image quality.

## Notes

This submission is made to the special issue for OXMI 2025.
